# Supplementary material for: Bell’s Palsy and COVID-19 Vaccines: A Systematic Review and Meta-Analysis
Source: Vaccines (Basel). 2023 Jan 20;11(2):236. doi: 10.3390/vaccines11020236 (PMC9961047; doi:10.3390/vaccines11020236)
Supplement: Supplementary file 1 [file vaccines-11-00236-s001.zip › Supplementary (1)_12_26_2022.pdf]

Table S7 represents the main clinical and pathological characteristics of the included patients.

| Study ID | Study design | Number of patients | Age (years) | Gender | Main signs and symptoms | Onset of facial palsy | Type of vaccine | Number of doses | Diagnostic tool | Result of diagnosis | Comorbidities | Treatment | Follow up | Prognosis |
|---|---|---|---|---|---|---|---|---|---|---|---|---|---|---|
| Cellina 2022 (1) | Case report | 1 | 35 | Female | left facial droop, left laterocervical pain and stiffness, slight difficulty in eating and speaking, inability to close ipsilateral eye, not holding liquids properly in mouth | 26 hours after her first dose | mRNA-1273 (Moderna) | 1 | CN examination confirmed by contrast-enhanced brain MRI | Isolated left LMN facial nerve palsy (Bell's palsy) | None | 50 mg/day prednisone for two weeks | 19 days | Complete resolution of symptoms after 19 days |

| | | | | | | | | | | | | | | |
|---|---|---|---|---|---|---|---|---|---|---|---|---|---|---|
| Cirillo 2022 / Colella 2021 (2,3) | Case report | 1 | 37 | Male | Flattening of labial-buccal sulcus ipsilaterally, forehead skin, and nasolabial fold, left-sided facial droop and paresis, moderate Bell's sign, Lagophthalmos, and labial weakness | 5 days after his first dose | BNT162b2 (Pfizer-BioNTech) | 1 | CN examination and EMG | Bell's palsy | None | 50 mg/day prednisone<br>Eye treatment | 17 months | Clinical signs progressed to full paralysis with pain in the first two weeks (worse symptoms)<br>Pain and facial weakness improvement with lagophthalmos gone after 8 months.<br>Complete recovery after 14 months |

| | | | | | | | | | | | | | | |
|---|---|---|---|---|---|---|---|---|---|---|---|---|---|---|
| Manea 2022 (4) | Case report | 1 | 29 | Male | Diplopia and facial nerve palsy | 6 days after his first dose | BNT162b2 (Pfizer-BioNTech) | 1 | CN examination and Gadolinium-enhanced brain MRI | On examination: Bell's palsy in addition to partial oculomotor (III) and abducent (IV) palsies, as well as hypoesthesia in V2 and V3 of trigeminal (V) nerve distribution.<br>MRI showed diffuse Gadolinium enhancement in labyrinthic and intracanalicular portions of facial nerve and in the intracisternal canals of oculomotor and left trigeminal nerves | None | 1 g/day methylprednisolone for 5 days | 2 months | Improvement of symptoms one month later with persistence of minimal left facial palsy |
| Martin-Villares 2022 (5) | Case report | 1 | 34 | Women | Facial pain and palsy,<br>H/O right bell's palsy during pregnancy 9 years ago | 2 days after her first dose | mRNA-1273 (Moderna) | 1 | MRI and nerve conduction studies | No relevant findings on MRI but nerve conduction studies showed axonal facial neuropathy | None | Deflazacort 30 mg/day, eye support and facial rehabilitation | 20 days | Improvement of symptoms started after 10 days and reached full recovery after 20 days |
| Mussatto 2022 (6) | Case report | 1 | 60 | Male | Inability to close his left eye completely lagophthalmos, and left facial droop and weakness | 42 hours after his first dose | BNT162b2 (Pfizer-BioNTech) | 2 | CN examination | Exposure keratopathy and uncomplicated left-sided bell's palsy | Stage 3 CKD, HIV, and pre-diabetic | Prednisone 60 mg/day for 6 days. valacyclovir 1000 mg 3 times/day for 7 day and eye support | 90 days | Significant improvement of symptoms after 15 days and complete resolution after 90 days |

| | | | | | | | | | | | | | | |
|---|---|---|---|---|---|---|---|---|---|---|---|---|---|---|
| Poudel 2022 (7) | Case report | 1 | 17 | Female | Inability to close her left eye in addition to blurry vision, left-sided facial muscle weakness | 24 hours after her vaccine dose | mRNA-1273 (Moderna) | NA | CN examination | Isolated right facial nerve palsy (Bell's palsy) | None | Prednisolone | >2 weeks | Excellent improvement of symptoms after 2 weeks and no weakness in the last follow up |
| Shalabi 2022 (8) | Case report | 1 | 41 | Male | Hoarseness and pain on the right shoulder, dysphagia, right hearing loss, dizziness, vertical diplopia, and right facial weakness | After a week of his first dose | BNT162b2 (Pfizer-BioNTech) | 1 | CN examination, chest and neck CT, audiogram, MRI, and a LP | On examination: right facial paresthesia and palsy, mild hoarseness, right 4th cranial nerve palsy, and right vocal cord paralysis. CT showed swollen right supraventricular LN. left sensorineural hearing impairment and right severe mixed hearing impairment on audiogram. MRI showed enhancement in the right auditory canal involving 7th and 8th cranial nerves. LP revealed increased proteins and CSF leukocytosis | None | IV methylprednisolone 1000 mg/day for 5 days | NA | Gradual improvement in all symptoms and resolution of the swollen lymph node |

| | | | | | | | | | | | | | |
|---|---|---|---|---|---|---|---|---|---|---|---|---|---|
| Zhang 2022 (9) | Case report | 1 | 38 | Female | Tongue numbness, right facial droop<br>H/O Bell's palsy 10 years ago | 7 days after her first dose | BNT162b2 (Pfizer-BioNTech) | 1 | CN examination | Bell's palsy | None | Prednisone for a week | NA | Slow improvement in symptoms |
| Burrows 2021 (10) | Case report | 1 | 61 | Male | Two different episodes of Bell's palsy after the first and the second doses of the vaccine<br>First episode: incomplete eye closure, right-sided facial weakness, and no forehead movement<br>Second episode: incomplete left eye closure, more severe left-sided facial palsy, dysphagia, and dribbling | 5 hours after his first dose.<br><br>2 days after his second dose | BNT162b2 (Pfizer-BioNTech) | 2 | Head CT and CN examination | Nothing remarkable on CT scan<br><br>Two episodes of facial nerve palsy (Bell's palsy) | insulin-dependent type 2 diabetes, hypercholesterolemia, and hypertension | 60 mg prednisolone for the first episode.<br><br>Another 60 mg prednisolone for 7 days for the second episode | 8 weeks | Complete recovery of the first episode.<br><br>Almost returned to normal after 2 weeks of the second episode |

| | | | | | | | | | | | | | | |
|---|---|---|---|---|---|---|---|---|---|---|---|---|---|---|
| Censcak 2021(11) | Case report | 1 | 42 | male | Unsteady gait, paresthesia of soles, right-sided muscle weakness, weak knees, right-sided lagophthalmos, and lumbalgia | 2 weeks after his first dose | BNT162b2 (Pfizer-BioNTech) | 1 | Neurological examination, LP, MRI, nerve conduction studies, and EMG | Acute demyelinating polyneuropathy (Guillan-Barré syndrome) | Bronchial asthma | IVIG 2 g/Kg for 5 days and rehabilitation | NA | All symptoms improved but mild facial weakness persisted |
| Ercoli 2021 (12) | Case report | 1 | 41 | Male | Difficulty to blink and move facial muscles after the first dose. respiratory impairment, swollen tongue, right-sided weakness, and left-sided facial hypoesthesia after the second dose | A few minutes after his first dose.<br><br>A couple of weeks after his second dose | NA | 2 | Neurological examination, head CT, and MRI | Normal CT and MRI. Diagnosed as functional neurological disorder (FND) | None | None | ≥2 weeks | Resolution of all symptoms spontaneously after 40 minutes of the first episode<br><br>Sensory disturbance disappeared after 2 weeks of the second episode. |

| | | | | | | | | | | | | | |
|---|---|---|---|---|---|---|---|---|---|---|---|---|---|
| Finsterer, 2021 (13) | Case report | 1 | 32 | Male | Dysphagia, paresthesia of foot soles and hand palms, bilateral muscle weakness, bilateral frontal and nuchal headache, sensory disturbance of tongue tip, and abnormal reflexes<br>H/O GBS | 8 days after his first dose | Vector based vaccine | 1 | Neurological examination,<br>urine toxicology, MRI, LP, and nerve conduction studies | Examination showed right-sided peripheral facial palsy.<br>Urine toxicology was positive for cannabinoids.<br>MRI showed nonspecific T2-hyperintensities in the white matter.<br>Slowed nerve conduction.<br>Diagnosed as GBS. | NA | IVIG 0.4 g/Kg and plasmapheresis | >34 days | Facial palsy slightly improved after 13 days but other complications occurred after nosocomial infection due to prolonged hospital admission |
| Iftikhar 2021 (14) | Case report | 1 | 36 | Male | Inability to close his left eye completely, left-sided facial weakness, numbness and weakness of his left arm, and difficulty in eating and speaking | After 1 day of his second dose | mRNA-1273 (Moderna) | 2 | CN examination, CT, and MRI | Isolated LMN palsy of the facial nerve on examination (Bell’s palsy)<br><br>CT and MRI findings were unremarkable | None | 60 mg/day oral prednisolone for one week and eye support | 2 weeks | Facial symptoms improved but still present and arm completely recovered |

| | | | | | | | | | | | | | |
|---|---|---|---|---|---|---|---|---|---|---|---|---|---|
| Introna 2021 (15) | Case report | 1 | 62 | Male | Bilateral facial weakness, visual discomfort, distal paresthesia, urinary retention, proximal ascending tetraparesis, and dysphagia | 13 days after his first dose | CHAdOx1 (AstraZeneca) | 1 | Neurological examination, fundoscopy, LP, and electrophysiological studies | CSF showed high opening pressure and albumin-cytologic dissociation. Fundoscopy showed severe bilateral optic disc edema. Diagnosed as GBS | Hypertension | IVIG 2 g/Kg for 5 days and rehabilitation | >4 weeks | Neurological symptoms improved after 7 days of IVIG, but the bilateral facial weakness persisted<br><br>The patient was bedridden after 4 weeks |
| Ish 2021 (16) | Case report | 1 | 50 | Male | Lagophthalmos, loss of forehead wrinkling, difficulty closing his right eye associated with redness and watering, conjunctival congestion, lower lid ectropion, and drooping of right mouth angle. | NA | Bharat Biotech (COVAXIN) | 2 | CN examination | Right LMN palsy of the facial nerve (Bell's palsy) | None | Oral prednisolone 1 mg/kg for 2 weeks and eye treatment | >10 days | Improvement of all symptoms after 10 days |

| | | | | | | | | | | | | | |
|---|---|---|---|---|---|---|---|---|---|---|---|---|---|
| Mason 2021 (17) | Case report | 1 | 35 | Female | First episode: inability to close her right eye, right-sided facial weakness, mild right arm weakness, occipital headaches, and difficulties in speech and swallowing Second episode: inability to close her eyes bilaterally, left-sided facial weakness, worsening of dysphagia and dysarthria, and inability to raise eyebrows or smile | 4 weeks after her vaccine dose for the first episode. 4 days after treatment for the second episode | mRNA-1273 (Moderna) | NA | MRI, CTA, LP, EMG, nerve conduction studies, and neurological examination | MRI and CTA were unremarkable. EMG and nerve conduction: 70% axonal loss of the left facial nerve when compared to right facial nerve | Migraine | 500 mg of IV methylprednisolone twice daily for 3 days and 400 mg of acyclovir 4 times/day for her first episode<br><br>Another 500 mg IV methylprednisolone twice daily and 400 mg of acyclovir 4 times/day after her second episode | >1 week | Remarkable improvement after 3 days of the second treatment |

| | | | | | | | | | | | | | | |
|---|---|---|---|---|---|---|---|---|---|---|---|---|---|---|
| Mousquer 2021 (18) | Case report | 1 | 78 | Female | Instability, malaise, nausea, pain in auditory canal, decrease right-sided facial mobility, vesicles and crusted lesions on concha of right ear, left horizontal rotatory nystagmus, gait instability, right sensorineural hearing loss | 5 days after her vaccine dose | BNT162b2 (Pfizer-BioNTech) | NA | Neurological examination | Right peripheral facial paralysis due to reactivation of Varicella-zoster virus (Ramsy Hunt syndrome) | Untreated atrial hypertension and H/O childhood poliomyelitis with lower limb sequelae and | NA | >2 weeks | After 2 weeks, slight improvement of the facial paralysis while the other symptoms persisted |
| Nasuelli 2021 (19) | Case report | 1 | 59 | Male | bilateral facial palsy, global areflexia, gait ataxia, and distal paresthesia in all limbs | 16 days after his vaccine dose | CHAdOx1 (AstraZeneca) | NA | Neurological examination, LP, EMG, and MRI | GBS | Hyperuricemia and hypertension | IVIG of 0.4 mg/Kg for 5 days and physiotherapy | >10 days after treatme nt | Slow improvement after 5 days of treatment while worsening of electrophysiological signs after 10 days |
| Nishizawa 2021 (20) | Case report | 1 | 62 | Female | Significant right-sided facial paralysis, right facial droop, incomplete eye closure | 20 days after his vaccine dose | Ad26.COV2.S (Johnson & Johnson) | 1 | Neurological examination, CT, and MRI | MRI and CT findings were unremarkable while the examination showed right-sided Bell's palsy | Hypertension, hyperlipidemia, and type 2 DM | NA | NA | NA |

| | | | | | | | | | | | | | | |
|---|---|---|---|---|---|---|---|---|---|---|---|---|---|---|
| Obermann 2021 (21) | Case report | 1 | 21 | Female | Facial muscle paralysis | 2 days after her first dose | BNT162b2 (Pfizer-BioNTech) | 1 | Neurological examination and MRI | MRI was unremarkable but examination revealed right-sided Bell's palsy | NA | Oral prednisolone 100 mg/day for 5 days and eye ointment | >3 days | After 3 days, lid closure returned but without improvement of her lower facial muscles |
| Pothiawala 2021(22) | Case report | 1 | 46 | Male | Tinnitus of right ear, loss of taste sensation, loss of right naso-labial fold, right-sided facial droop, and inability to close his right eye completely | 10 to 13 days after his second dose | mRNA-1273 (Moderna) | 2 | Neurological examination | Isolated right LMN facial nerve palsy (Bell's palsy) | None | Acyclovir and prednisone | NA | NA |
| Repajic 2021 (23) | Case report | 1 | 57 | Female | Facial droop, loss of taste sensation, inability to close her left eye completely, soreness behind left ear above her jaw, and hyperacusis. H/O three previous Bell's palsies treated with corticosteroids | <36 hours after her second dose | BNT162b2 (Pfizer-BioNTech) | 2 | CN examination | Isolated left facial nerve palsy (Bell's palsy) | Corticosteroid-induced hypertension | Prednisone and an antiviral agent | >14 days | Symptoms started to improve after 14 days |

| | | | | | | | | | | | | | | |
|---|---|---|---|---|---|---|---|---|---|---|---|---|---|---|
| Yu 2021 (24) | Case report | 1 | 36 | Female | Inability to close her eye completely, eye discomfort, right-sided facial weakness, and forehead wrinkles disappearance. H/O Bell's palsy 18 years ago | 2 days after his first dose | Sinovac Life Science vaccine | 1 | CN examination | Isolated right facial nerve palsy (Bell's palsy) | None | Prednisone 40 g/day for one week and eye support | 54 days | Symptoms began to improve after 14 days and completely resolved after 54 days |
| Rahman 2022) (25) | Case series | 53 | 50.1±15.2* | Male: 27 (50.9%) Female: 26 (49.1%) | Bell's palsy | NA | BNT162b2 (Pfizer-BioNTech): 27 (50.9%)<br><br>Sinovac Life Science vaccine: 21 (39.6%)<br><br>CHAdOx1 (AstraZeneca): 5 (9.4%) | One dose: 33 (62.3%)<br><br>Two doses: 20 (37.7%) | NA | Bell's palsy | Hypertension: 18 (34%)<br><br>Diabetes: 15 (28.3%)<br><br>Heart disease: 5 (9.4%) | NA | 21 days after vaccine | NA |

| | | | | | | | | | | | | | |
|---|---|---|---|---|---|---|---|---|---|---|---|---|---|
| Andreozzi 2022) (26) | Case series | Case 1 | 59 | Female | Lower back pain, complete facial diplegia, bell's phenomena, bilateral facial weakness, lagophthalmos, abnormal limb reflexes, mild dysarthria, paresthesia, and inability to raise both eyebrows, whistle, and smile | 15 days following her dose | CHAdOx1 (AstraZeneca) | 1 | Neurological examination, LP, CT, and electrophysiological studies | LP: CSF albuminocytologic dissociation. Electrophysiological studies and examination were consistent with GBS with bifacial weakness with paresthesia | Hashimoto thyroiditis (acquired hypothyroidism) | IVIG of 0.4 mg/Kg for 5 days | 2 days | Improvement of symptoms started 2 days after the treatment |
| | | Case 2 | 43 | male | Facial pain and numbness, lower limb and hand paresthesia, weakness of eye closure, left-sided facial paralysis, and lower limb hyporeflexia | 7 days after his first dose | CHAdOx1 (AstraZeneca) | 1 | Neurological examination, LP, and electrophysiological studies | LP: CSF albuminocytologic dissociation. Electrophysiological studies and examination were consistent with GBS with bifacial weakness with paresthesia | None | IVIG of 0.4 mg/Kg for 5 days | 8 days | Excellent improvement of facial weakness and paresthesia |
| Castiglione 2022 (27) | Case series | Case 1 | 56 | Female | Distal paresthesia in all limbs and bilateral facial weakness | 19 days after her first dose | Sputnik V | 1 | Neurological examination, LP, electrophysiological studies, and MRI | Bilateral facial palsy with paresthesia variant of GBS | NA | IVIG | >8 days | Symptom improvement |

| | | | | | | | | | | | | | | |
|---|---|---|---|---|---|---|---|---|---|---|---|---|---|---|
| | | Case 2 | 55 | Male | Distal paresthesia in all limbs, bilateral facial palsy, and reduced patellar and bicipital reflexes | 28 days after his first dose | Sputnik V | 1 | Neurological examination, LP, electrophysiological studies, and MRI | Bilateral facial palsy with paresthesia variant of GBS | NA | IVIG | >28 days | Symptom improvement |
| | | Case 3 | 87 | Male | Hyporeflexia and distal paresthesia in all limbs and bilateral facial weakness | 17 days after his first dose | Sputnik V | 1 | Neurological examination, LP, electrophysiological studies, and MRI | Bilateral facial palsy with paresthesia variant of GBS | NA | None | 3 days | Death after 3 days due to sudden arrhythmia prior to treatment |
| | | Case 4 | 50 | Male | General areflexia and paresthesia and bilateral facial palsy | 20 days after his first dose | CHAdOx1 (AstraZeneca) | 1 | Neurological examination, LP, electrophysiological studies, and MRI | Bilateral facial palsy with paresthesia variant of GBS | NA | IVIG | >18 days | Symptom improvement |
| | | Case 5 | 39 | Male | Paresthesia in both feet, altered tase, and sever bilateral facial weakness | 10 days after his first dose | Sputnik V | 1 | Neurological examination, LP, electrophysiological studies, and MRI | Bilateral facial palsy with paresthesia variant of GBS | NA | IVIG | >23 days | Symptom improvement |
| | | Case 6 | 42 | Male | Generalized hyporeflexia, paresthesia, radicular pain, and bilateral facial weakness | 28 days after his first dose | CHAdOx1 (AstraZeneca) | 1 | Neurological examination, LP, electrophysiological studies, and MRI | Bilateral facial palsy with paresthesia variant of GBS | NA | IVIG | >10 days | Symptom improvement |

| | | | | | | | | | | | | | |
|---|---|---|---|---|---|---|---|---|---|---|---|---|---|
| | | Case 7 | 52 | Female | Paresthesia in all limbs, absent tendon reflexes, and bilateral facial weakness | 13 days after her first dose | CHAdOx1 (AstraZeneca) | 1 | Neurological examination, LP, electrophysiological studies, and MRI | Bilateral facial palsy with paresthesia variant of GBS | NA | Plasma exchange | >20 days | Symptom improvement |
| | | Case 8 | 43 | Male | Reduced deep tendon reflexes, radicular pain, paresthesia, and bilateral facial weakness | 13 days after his second dose | Sputnik V | 2 | Neurological examination, LP, electrophysiological studies, and MRI | Bilateral facial palsy with paresthesia variant of GBS | NA | IVIG | >53 days | Symptom improvement |
| | | Case 9 | 65 | Male | Numbness in hands and feet, leg cramping pain, and severe bilateral facial weakness | 13 days after his second dose | CHAdOx1 (AstraZeneca) | 2 | Neurological examination, LP, electrophysiological studies, and MRI | Bilateral facial palsy with paresthesia variant of GBS | NA | IVIG | >7 days | Symptom improvement |
| Hamid 2022 (28) | Case series | Case 1 | 23 | Male | Headache, lagophthalmos, and right facial muscle weakness | Same day of his first dose | BNT162b2 (Pfizer-BioNTech) | 1 | Neurological examination, LP, and MRI | MRI: diffuse contrast enhancement in mastoid, tympanic, and labyrinthine portions of right facial nerve.<br><br>Diagnosed as right-sided Bell's palsy | None | Prednisolone | >2 months | NA |

| | | | | | | | | | | | | | |
|---|---|---|---|---|---|---|---|---|---|---|---|---|---|
| | | Case 2 | 27 | Male | Left-sided cervicogenic headache, drooping of mouth, and facial droop | 2 months after his first dose | BNT162b2 (Pfizer-BioNTech) | 1 | Neurological examination and MRI | MRI: non-mass-like and linear gadolinium enhancement in internal auditory canal portion of left facial nerve.<br><br>Diagnosed as left-sided Bell's palsy | None | Oral prednisone | >18 days | Incomplete response without returning to normal |
| | | Case 3 | 20 | Female | Headache, binocular diplopia, lateral gaze limitation in right eye, and right facial muscle weakness | 5 days after her second dose | BNT162b2 (Pfizer-BioNTech) | 2 | Neurological examination and MRI | MRI: non-nodular and vivid gadolinium enhancement in labyrinth portion of right facial nerve reaching the geniculate ganglion<br><br>Diagnosed as right-sided Bell's palsy and right lateral rectus palsy | None | IV prednisolone 1 g/day for 10 days | >5 days | Full symptomatic resolution |
| Mirmosayyeb 2022 (29) | Case series | Case 1 | 27 | Female | SCM pain, upper left lip shifting to the right, inability to close her left eye completely | 5-6 days after her first dose | Sputnik V | 1 | Neurological examination and MRI | MRI was normal but examination revealed left-sided Bell's palsy | None | Prednisolone 50 mg/day for one week<br>Virabex and valacyclovir 1000 mg 2 times/day | 10 days | Symptoms resolved with persistence of mild pain in the SCM area |

| | | | | | | | | | | | | | | |
|---|---|---|---|---|---|---|---|---|---|---|---|---|---|---|
| | | Case 2 | 58 | Male | Left-sided mouth droop, drooling, slurred speech, left-sided facial weakness, decreased taste sensation, inability to close his left eye, and tearing | 9-10 days after his first dose | Sputnik V | 1 | Neurological examination | Left-sided Bell's palsy | Controlled DM | Prednisolone 50 mg for a week\ Valacyclovir 1000 mg 2 times/day | 1 week | Complete recovery |
| Correa 2021 (30) | Case series | Case 2 (other cases do not involve facial nerve) | 42 | Male | Left lagophthalmos, forehead muscle paresis, labial weakness, left facial muscle weakness | 7 days after his first dose | CHAdOx1 (AstraZeneca) | 1 | Neurological examination and MRI | MRI showed gadolinium enhancement in labyrinth and canalicular portions of left facial nerve reaching the geniculate ganglion diagnosed as left-sided Bell's palsy | None | Oral prednisone 60 mg/day for 7 days | 7 days | Complete resolution of symptoms |
| James 2021 (31) | Case series | case 1 (other cases do not involve facial nerve) | 60 | Male | LMN Bifacial palsy, numbness of fingers and toes, proximal weakness in all limbs, and absent lower limb reflexes | 11-14 days after his first dose | CHAdOx1 (AstraZeneca) | 1 | Neurological examination, nerve conduction study, LP, and MRI | Sensorimotor axonal neuropathy (GBS) | COPD | IGIV of 2g/Kg for 5 days | 3 weeks | The progression of the disease stopped, and some symptoms showed improvements |

| | | | | | | | | | | | | | |
|---|---|---|---|---|---|---|---|---|---|---|---|---|---|
| Shemer 2021 (32) | Case series | Case 1 | 86 | Female | Facial weakness, asymmetrical smile, corneal erosion on left eye, and inability to close his left eye completely | 14 days after her first dose | BNT162b2 (Pfizer-BioNTech) | 1 | Neurological examination | Left peripheral facial palsy (Bell's palsy) | Hypertension | Oral steroids and eye support | NA | NA |
| | | Case 2 | 78 | Female | Left facial weakness, ear pain, and tinnitus | 5 days after her second dose | BNT162b2 (Pfizer-BioNTech) | 2 | Neurological examination | Left peripheral facial palsy (Bell's palsy) | Cardiac pacemaker | Conventional treatment | NA | Development of periauricular rash and sensorineural hearing loss bilaterally 2 days after hospital discharge treated with an antimicrobial |
| | | Case 3 | 79 | Male | Right-sided herpes zoster opthalmicus after first dose, inability to close his right eye completely and asymmetrical smile after his second dose | 2 days after second dose | BNT162b2 (Pfizer-BioNTech) | 2 | NA | Right peripheral facial palsy (Bell's palsy) | Asthma and hypertension | Oral steroids | NA | NA |
| | | Case 4 | 69 | Female | Asymmetry in the face | 3 days after her first dose | BNT162b2 (Pfizer-BioNTech) | 1 | Neurological examination | Right peripheral facial palsy (Bell's palsy) | None | Steroidal treatment and eye support | NA | NA |

| | | | | | | | | | | | | | |
|---|---|---|---|---|---|---|---|---|---|---|---|---|---|
| | | Case 5 | 73 | Female | Asymmetry in the face | 12 days after her first dose | BNT162b2 (Pfizer-BioNTech) | 1 | Neurological examination | Left peripheral facial palsy (Bell's palsy) | Dyslipidemia | Steroidal treatment and eye support | NA | NA |
| | | Case 6 | 77 | Male | Asymmetry in the face | 22 days after first his dose/ 1 day after second dose | BNT162b2 (Pfizer-BioNTech) | 2 | Neurological examination | Left peripheral facial palsy (Bell's palsy) | Stroke, prostate cancer, and hypertension | Steroidal treatment and eye support | NA | NA |
| | | Case 7 | 64 | Male | Asymmetry in the face | 7 days after his first dose | BNT162b2 (Pfizer-BioNTech) | 1 | Neurological examination | Left peripheral facial palsy (Bell's palsy) | None | Steroidal treatment and eye support | NA | NA |
| | | Case 8 | 51 | Male | Asymmetry in the face | 30 days after his first dose/ 9 days after second dose | BNT162b2 (Pfizer-BioNTech) | 2 | Neurological examination | Right peripheral facial palsy (Bell's palsy) | Obstructive apnea, Meniere's disease, and hypertension | Steroidal treatment and eye support | NA | NA |
| | | Case 9 | 35 | Male | Asymmetry in the face | 4 days after his first dose | BNT162b2 (Pfizer-BioNTech) | 1 | Neurological examination | Left peripheral facial palsy (Bell's palsy) | None | Steroidal treatment and eye support | NA | NA |

Abbreviations: CN = cranial nerve, MRI = Magnetic resonance imaging, LMN = lower motor neuron, EMG = Electromyography, H/O = history of, GBS = Guillan-Barré syndrome, CSF = cerebrospinal fluid, CKD = chronic kidney disease, IVIG = intravenous immunoglobulins, HIV = human immunodeficiency virus, CT = computed tomography, LP = lumbar puncture, LN = lymph node, DM = diabetes mellitus, CTA = Computed tomography angiography, NA = not available.

Table S8 represents the characteristics of cohort, case-control, cross-sectional studies, and self-controlled case series included our systematic review

| Study ID | Location | Group age | Period | Study design | Source of data |
|---|---|---|---|---|---|

| | | | | | |
|---|---|---|---|---|---|
| Bardenheier 1 2021 (33) | US | Adults | December 2020 till February 2021 | Cohort study | Registry |
| Bardenheier 2 2021 (34) | US | Adults | December 2020 till March 2021 | Cohort study | Registry |
| Clemens 2022 (35) | Brazil | Adults | April 2021 till September 2021 | Cohort study | Hospital records |
| El-Shitany 2021 (36) | Saudi Arabia | Adults | December 2021 till February 2022 | cross-sectional study | Online survey |
| El-Shitany 2022 (37) | Saudi Arabia | Adults | January 10, 2021, till January 21 2021 | cross-sectional study | Online survey |
| Frontera 2021 (38) | US | Adults | January 2021 till June 2021 | Retrospective cohort study | US Vaccine Adverse Event Reporting System (VAERS) |
| Huh 2021 (39) | Korea | All ages | Up to June 2020 | Cohort study | Registry |
| Kant 2022 (40) | Netherlands | Adolescents and adults | February 2021 till August 2021 | Prospective cohort study | Online questionnaires |
| Koh 2021 (41) | Singapore | Adults | December 2020 till April 2021 | Prospective cohort study | Registry |
| Li 2022 (42) | UK and Spain | Adults | December 2020 till June 2021 | Cohort study | Registry |
| Mohammed 2021 (43) | Saudi Arabia | Adults | February 2021 till March 2021 | Cross-sectional study | Online survey |

| | | | | | |
|---|---|---|---|---|---|
| Nasreen 2022 (44) | Canada | All ages | Up to December 2020 | Cohort study | Registry |
| Noseda 2021 (45) | NR | Adults | Up to May 2021 | Case-control study | VigiBase (WHO) |
| Patone 2021 (46) | UK | Adults | December 2020 till May 2021 | self-controlled case series | Registry |
| Shibli 2021 (47) | Israel | Adults | December 2020 till April 2021 | Retrospective cohort study | Registry |
| Tsun Lai 2022 (48) | Hong Kong | Adolescents (12-18y) | Up to September 2021 | Retrospective cohort study | Registry |
| Walker 2022 (49) | UK | Adults | July 2020 till July 2021 | self-controlled case series | Registry |
| Wong 2021 (50) | Hong Kong | Adults | February 2021 till September 2021 | Retrospective cohort study | Registry |
| Xu 2022 (51) | US | All ages | Up to December 2020 | Retrospective cohort study | Registry |
| Yuk Fai Wan1 2022 (51) | Hong Kong | Adults | February 2021 till May 2021 | Nested case-control study | Registry |
| Yuk Fai Wan2 2022 (52) | Hong Kong | Adults | March 2021 till July 2021 | Nested case-control study | Registry |

NR: Not reported

***References***


1. Cellina M, D'Arrigo A, Floridi C, Oliva G, Carrafiello G. Left Bell's palsy following the first dose of mRNA-1273 SARS-CoV-2 vaccine: A case report. Clin Imaging. 2022 Feb;82:1–4.
2. Cirillo N, Orlandi M, Colella G. Patients with post-COVID-19 vaccination facial palsy: To boost or not to boost? Transl Neurosci. 2022 Jan 1;13(1):236–9.
3. Colella G, Orlandi M, Cirillo N. Bell's palsy following COVID-19 vaccination. J Neurol. 2021 Oct;268(10):3589–91.
4. Manea MM, Dragoș D, Enache I, Sirbu AG, Tuta S. Multiple cranial nerve palsies following COVID-19 vaccination-Case report. Acta Neurol Scand. 2022 Feb;145(2):257–9.
5. Martin-Villares C, Vazquez-Feito A, Gonzalez-Gimeno MJ, de la Nogal-Fernandez B. Bell's palsy following a single dose of mRNA SARS-CoV-2 vaccine: a case report. J Neurol. 2022 Jan;269(1):47–8.
6. Mussatto CC, Sokol J, Alapati N. Bell's palsy following COVID-19 vaccine administration in HIV+ patient. Am J Ophthalmol Case Rep. 2022 Mar;25:101259.
7. Poudel S, Nepali P, Baniya S, Shah S, Bogati S, Nepal G, et al. Bell's palsy as a possible complication of mRNA-1273 (Moderna) vaccine against COVID-19. Ann Med Surg (Lond). 2022 Jun;78:103897.
8. Shalabi F, Lossos A, Karussis D. A case report of unilateral cervical lymphadenopathy and multiple cranial neuropathies following mRNA-COVID-19 vaccination. BMC Neurol. 2022 Sep 26;22(1):369.
9. Zhang H, Sanchez Gomez D, Repajic M, Liu AK. Another Case of Bell's Palsy Recurrence After Pfizer-BioNTech COVID-19 Vaccination. Cureus. 2022 Jul 28;14(7):e27422.
10. Burrows A, Bartholomew T, Rudd J, Walker D. Sequential contralateral facial nerve palsies following COVID-19 vaccination first and second doses. BMJ Case Rep. 2021 Jul 19;14(7).
11. Čenščák D, Ungermann L, Štětkářová I, Ehler E. Guillan-Barré Syndrome after First Vaccination Dose against COVID-19: Case Report. Acta Medica (Hradec Kralove). 2021;64(3):183–6.
12. Ercoli T, Lutzoni L, Orofino G, Muroni A, Defazio G. Functional neurological disorder after COVID-19 vaccination. Neurol Sci. 2021 Oct;42(10):3989–90.
13. Finsterer J. Exacerbating Guillain-Barré Syndrome Eight Days after Vector-Based COVID-19 Vaccination. Case Rep Infect Dis. 2021 May 8;2021:3619131.
14. Iftikhar H, Noor SMU, Masood M, Bashir K. Bell's Palsy After 24 Hours of mRNA-1273 SARS-CoV-2 Vaccine. Cureus. 2021 Jun 26;13(6):e15935.
15. Introna A, Caputo F, Santoro C, Guerra T, Ucci M, Mezzapesa DM, et al. Guillain-Barré syndrome after AstraZeneca COVID-19-vaccination: A causal or casual association? Clin Neurol Neurosurg. 2021 Sep;208:106887.
16. Ish S, Ish P. Facial nerve palsy after COVID-19 vaccination - A rare association or a coincidence. Indian J Ophthalmol. 2021 Sep;69(9):2550–2.
17. Mason MC, Liaqat A, Morrow J, Basso R, Gujrati Y. Bilateral Facial Nerve Palsy and COVID-19 Vaccination: Causation or Coincidence? Cureus. 2021 Aug 31;13(8):e17602.

18. Mousquer GT, Peres A, Fiegenbaum M. Pathology of TB/COVID-19 Co-Infection: The phantom menace. Tuberculosis (Edinb). 2021 Jan;126:102020.

19. Nasuelli NA, De Marchi F, Cecchin M, De Paoli I, Onorato S, Pettinaroli R, et al. A case of acute demyelinating polyradiculoneuropathy with bilateral facial palsy after ChAdOx1 nCoV-19 vaccine. Neurol Sci. 2021 Nov;42(11):4747–9.

20. Nishizawa Y, Hoshina Y, Baker V. Bell's palsy following the Ad26.COV2.S COVID-19 vaccination. QJM. 2021 Nov 13;114(9):657–8.

21. Obermann M, Krasniqi M, Ewers N, Fayad J, Haeberle U. Bell's palsy following COVID-19 vaccination with high CSF antibody response. Neurol Sci. 2021 Nov;42(11):4397–9.

22. Pothiawala S. Bell's Palsy After Second Dose of Moderna COVID-19 Vaccine: Coincidence or Causation? Acta Med Litu. 2021 Aug 4;28(2):298–301.

23. Repajic M, Lai XL, Xu P, Liu A. Bell's Palsy after second dose of Pfizer COVID-19 vaccination in a patient with history of recurrent Bell's palsy. Brain Behav Immun Health. 2021 May;13:100217.

24. Yu B-Y, Cen L-S, Chen T, Yang T-H. Bell's palsy after inactivated COVID-19 vaccination in a patient with history of recurrent Bell's palsy: A case report. World J Clin Cases. 2021 Sep 26;9(27):8274–9.

25. Ab Rahman N, Lim MT, Lee FY, Lee SC, Ramli A, Saharudin SN, et al. Risk of serious adverse events after the BNT162b2, CoronaVac, and ChAdOx1 vaccines in Malaysia: A self-controlled case series study. Vaccine. 2022 Jul 30;40(32):4394–402.

26. Andreozzi V, D'arco B, Pagliano P, Toriello A, Barone P. Bilateral facial palsy after COVID-19 vaccination. Neurol Sci. 2022 Jul;43(7):4069–79.

27. Castiglione JI, Crespo JM, Lecchini L, Silveira FO, Luis MB, Cotti N, et al. Bilateral facial palsy with paresthesias, variant of Guillain-Barré syndrome following COVID-19 vaccine: A case series of 9 patients. Neuromuscul Disord. 2022 Jul;32(7):572–4.

28. Hamid R, Korkmazer B, Kochan Kizilkilic E, Arman GM, Ozogul M, Kargın OA, et al. Potential Association Between COVID-19 Vaccination and Facial Palsy: Three Cases With Neuroimaging Findings. Ear Nose Throat J. 2022 Jul 19;1455613221113818.

29. Mirmosayyeb O, Barzegar M, Rezaei M, Baharlouie N, Shaygannejad V. Bell's palsy after Sputnik V COVID-19 (Gam-COVID-Vac) vaccination. Clin Case Rep. 2022 Feb 23;10(2):e05468.

30. Corrêa DG, Cañete LAQ, Dos Santos GAC, de Oliveira RV, Brandão CO, da Cruz LCH. Neurological symptoms and neuroimaging alterations related with COVID-19 vaccine: Cause or coincidence? Clin Imaging. 2021 Dec;80:348–52.

31. James J, Jose J, Gafoor VA, Smita B, Balaram N. Guillain-Barré syndrome following ChAdOx1 nCoV-19 COVID-19 vaccination: A case series. Neurol Clin Neurosci. 2021 Sep;9(5):402–5.

32. Shemer A, Pras E, Einan-Lifshitz A, Dubinsky-Pertzov B, Hecht I. Association of COVID-19 Vaccination and Facial Nerve Palsy: A Case-Control Study. JAMA Otolaryngol Head Neck Surg. 2021 Aug 1;147(8):739–43.

33. Bardenheier BH, Gravenstein S, Blackman C, Gutman R, Sarkar IN, Feifer RA, et al. Adverse Events Following One Dose of mRNA COVID-19 Vaccination Among US Nursing Home Residents With and Without a Previous SARS-CoV-2 Infection. J Am Med Dir Assoc. 2021 Nov;22(11):2228–32.

34. Bardenheier BH, Gravenstein S, Blackman C, Gutman R, Sarkar IN, Feifer RA, et al. Adverse events following mRNA SARS-CoV-2 vaccination among U.S. nursing home residents. Vaccine. 2021 Jun 29;39(29):3844–51.

35. Clemens SAC, Fortaleza CMCB, Crowe M, Pollard A, Tasca KI, Grotto RMT, et al. Safety of the Fiocruz ChAdOx COVID-19 vaccine used in a mass vaccination campaign in Botucatu, Brazil. Vaccine. 2022 Nov 8;40(47):6722–9.

36. El-Shitany NA, Harakeh S, Badr-Eldin SM, Bagher AM, Eid B, Almukadi H, et al. Minor to Moderate Side Effects of Pfizer-BioNTech COVID-19 Vaccine Among Saudi Residents: A Retrospective Cross-Sectional Study. Int J Gen Med. 2021 Apr

19;14:1389–401.

37. El-Shitany NA, Bagher AM, Binmahfouz LS, Eid BG, Almukadi H, Badr-Eldin SM, et al. The Adverse Reactions of Pfizer BioNTech COVID-19 Vaccine Booster Dose are Mild and Similar to the Second Dose Responses: A Retrospective Cross-Sectional Study. Int J Gen Med. 2022 Aug 26;15:6821–36.

38. Frontera JA, Tamborska AA, Doheim MF, Garcia-Azorin D, Gezegen H, Guekht A, et al. Neurological Events Reported after COVID-19 Vaccines: An Analysis of VAERS. Ann Neurol. 2022 Mar 2;91(6):756–71.

39. Huh K, Kim YE, Radnaabaatar M, Lee DH, Kim DW, Shin SA, et al. Estimating Baseline Incidence of Conditions Potentially Associated with Vaccine Adverse Events: a Call for Surveillance System Using the Korean National Health Insurance Claims Data. J Korean Med Sci. 2021 Mar 8;36(9):e67.

40. Kant A, Jansen J, van Balveren L, van Hunsel F. Description of Frequencies of Reported Adverse Events Following Immunization Among Four Different COVID-19 Vaccine Brands. Drug Saf. 2022 Apr;45(4):319–31.

41. Koh JS, Hoe RHM, Yong MH, Chiew HJ, Goh Y, Yong KP, et al. Hospital-based observational study of neurological disorders in patients recently vaccinated with COVID-19 mRNA vaccines. J Neurol Sci. 2021 Nov 15;430:120030.

42. Li X, Raventós B, Roel E, Pistillo A, Martinez-Hernandez E, Delmestri A, et al. Association between covid-19 vaccination, SARS-CoV-2 infection, and risk of immune mediated neurological events: population based cohort and self-controlled case series analysis. BMJ. 2022 Mar 16;376:e068373.

43. Mohammed RA, Garout RM, Wahid S, Ayub F, Firas ZinAlddin LM, Sultan I. A Survey on the Side Effects of Pfizer/BioNTech COVID-19 Vaccine Among Vaccinated Adults in Saudi Arabia. Cureus. 2021 Nov 3;13(11):e19222.

44. Nasreen S, Calzavara A, Buchan SA, Thampi N, Johnson C, Wilson SE, et al. Background incidence rates of adverse events of special interest related to COVID-19 vaccines in Ontario, Canada, 2015 to 2020, to inform COVID-19 vaccine safety surveillance. Vaccine. 2022 May 26;40(24):3305–12.

45. Noseda R, Ripellino P, Ghidossi S, Bertoli R, Ceschi A. Reporting of Acute Inflammatory Neuropathies with COVID-19 Vaccines: Subgroup Disproportionality Analyses in VigiBase. Vaccines (Basel). 2021 Sep 14;9(9).

46. Patone M, Handunnetthi L, Saatci D, Pan J, Katikireddi SV, Razvi S, et al. Neurological complications after first dose of COVID-19 vaccines and SARS-CoV-2 infection. Nat Med. 2021 Dec;27(12):2144–53.

47. Shibli R, Barnett O, Abu-Full Z, Gronich N, Najjar-Debbiny R, Doweck I, et al. Association between vaccination with the BNT162b2 mRNA COVID-19 vaccine and Bell’s palsy: a population-based study. Lancet Reg Health Eur. 2021 Dec;11:100236.

48. Lai FTT, Chua GT, Chan EWW, Huang L, Kwan MYW, Ma T, et al. Adverse events of special interest following the use of BNT162b2 in adolescents: a population-based retrospective cohort study. Emerg Microbes Infect. 2022 Dec;11(1):885–93.

49. Walker JL, Schultze A, Tazare J, Tamborska A, Singh B, Donegan K, et al. Safety of COVID-19 vaccination and acute neurological events: A self-controlled case series in England using the OpenSAFELY platform. Vaccine. 2022 Jul 30;40(32):4479–87.

50. Wong CKH, Lau KTK, Xiong X, Au ICH, Lai FTT, Wan EYF, et al. Adverse events of special interest and mortality following vaccination with mRNA (BNT162b2) and inactivated (CoronaVac) SARS-CoV-2 vaccines in Hong Kong: A retrospective study. PLoS Med. 2022 Jun 21;19(6):e1004018.

51. Xu S, Hong V, Sy LS, Glenn SC, Ryan DS, Morrissette KL, et al. Changes in incidence rates of outcomes of interest in vaccine safety studies during the COVID-19 pandemic. Vaccine. 2022 May 20;40(23):3150–8.

52. Wan EYF, Chui CSL, Ng VWS, Wang Y, Yan VKC, Lam ICH, et al. mRNA (BNT162b2) COVID-19 vaccination increased risk of Bell's palsy: a nested case control and self-controlled case series study. Clin Infect Dis. 2022 Jun 8;
