# Supplementary material for: Bell’s Palsy and COVID-19 Vaccines: A Systematic Review and Meta-Analysis
Source: Vaccines (Basel). 2023 Jan 20;11(2):236. doi: 10.3390/vaccines11020236 (PMC9961047; doi:10.3390/vaccines11020236)
Supplement: Supplementary file 1 [file vaccines-11-00236-s001.zip › Supplemetary (2)_12_26_2022.pdf]

Table S1 shows quality Assessment of included Cases Reports using (JBI) Quality Assessment Tool.

Study ID	The Joanna Briggs Institute (JBI) Critical Appraisal checklist for Case Reports								Quality (Total Quality Score)	Risk Of Bias	Final Appraisal
	Were the patient's demographic characteristics clearly described?	Was the patient's history clearly described and presented as a timeline?	Was the current clinical condition of the patient on presentation clearly described?	Were diagnostic tests or assessment methods and the results clearly described?	Was the intervention(s) or treatment procedure(s) clearly described?	Was the post-intervention clinical condition clearly described?	Were adverse events (harms) or unanticipated events identified and described?	Does the case report provide takeaway lessons?			
Burrows 2021 [1]	YES	YES	YES	YES	YES	YES	NO	YES	88%	High	Include
Cellina 2022 [2]	YES	YES	YES	YES	YES	YES	NO	YES	88%	High	Include
Čenščák 2021 [3]	YES	YES	YES	YES	YES	YES	NO	YES	88%	High	Include
Cirillo 2022 [4]	NO	NO	YES	YES	YES	YES	NO	YES	62.50 %	Moderate	Include
Colella 2021 [5]	YES	YES	YES	NO	YES	YES	NO	YES	75%	High	Include
Ercoli 2021 [6]	Yes	YES	YES	YES	YES	YES	NO	YES	88%	High	Include
Finsterer 2021 [7]	Yes	YES	YES	YES	YES	YES	NO	YES	88%	High	Include
Iftikhar 2021 [8]	YES	YES	YES	YES	YES	YES	NO	YES	88%	High	Include
Introna 2021 [9]	YES	YES	YES	YES	YES	YES	NO	YES	88%	High	Include
Ish 2021 [10]	YES	NO	YES	NO	YES	YES	NO	YES	62.50 %	Moderate	Include
Manea 2021 [11]	YES	YES	YES	YES	YES	YES	NO	YES	88%	High	Include
Martin-Villares 2021 [12]	NO	YES	YES	YES	NO	YES	NO	YES	75%	High	Include
Mason 2021	YES	NO	YES	YES	YES	YES	NO	YES	75%	High	Include

[13]											
Mussatto 2021 [14]	YES	NO	YES	YES	YES	YES	NO	YES	75%	High	Include
Nasuelli 2021 [15]	YES	NO	YES	YES	YES	YES	NO	YES	75%	High	Include
Nishizawa 2021 [16]	YES	YES	YES	YES	NO	NO	NO	YES	62.50 %	Mode rate	Include
Obermann 2021 [17]	YES	YES	YES	YES	YES	YES	NO	YES	88%	High	Include
Pothiawala 2021 [18]	YES	YES	YES	NO	YES	NO	NO	YES	62.50 %	Mode rate	Include
Poudel 2022 [19]	YES	NO	YES	YES	YES	YES	NO	YES	75%	High	Include
Repajic 2021 [20]	YES	YES	YES	NO	YES	YES	NO	YES	75%	High	Include
Shalabi 2022 [21]	YES	NO	YES	YES	YES	YES	NO	YES	75%	High	Include
Yu 2021 [22]	YES	NO	YES	YES	YES	YES	NO	YES	75%	High	Include
Zhang 2022 [23]	YES	NO	YES	NO	YES	YES	NO	YES	62.50 %	Mode rate	Include

Low risk: 75-100%, **Moderate:** 50-74%, **High risk** <50%, **include** Moderate and Low risk of bias and **exclude** High risk of bias.

Table S2: shows quality Assessment of included Cases series studies using (NIH) Quality Assessment Tool.

	NIH Quality Assessment Tool for Case Series Studies Criteria Met									
Study ID	Was the study question or objective clearly stated?	Was the study population clearly and fully described, including a case definitio	Were the cases consecutive?	Were the subjects comparable?	Was the intervention clearly described?	Were the outcome measures clearly defined, valid, reliable, and implemented consisten	Was the length of follow-up adequate?	Were the statistical methods well-described?	Were the results well-described?	Quality (Total Quality Score)

		n?				tly across all study participa nts?				
Andreozzi 2022 [24]	YES	YES	NA	YES	YES	YES	NA	NO	YES	6 (Fair)
Castiglionea 2022 [25]	YES	YES	YES	YES	YES	YES	NA	NO	YES	7 (Good)
Correa 2021 [26]	YES	YES	NA	NO	YES	YES	NO	NO	YES	5 (Fair)
Hamid 2022 [27]	YES	YES	NA	NO	YES	YES	NA	NO	YES	6 (Fair)
James 2021 [28]	YES	YES	NA	YES	YES	YES	YES	NO	YES	7 (Fair)
Mirmosayyeb 2021 [29]	YES	YES	NA	NO	YES	YES	NO	NO	YES	5 (Fair)
Shemer 2021 [30]	YES	NO	YES	YES	NO	YES	NA	NO	YES	5 (Fair)

NA: Not Applicable, **Good**: Met 7-9 criteria, **Fair**: Met 4-6 Criteria, **Poor**: Met 0-3 Criteria, **JBI** = Joanna Briggs Institute.

Table S3 shows quality Assessment of included self-controlled Cases series studies using (NIH) Quality Assessment Tool.

Study ID	NIH Quality Assessment Tool for Case Series Studies Criteria Met									Quality (Total Quality Score)
	Was the study question or objective clearly stated?	Was the study populati on clearly and fully describe d, including a case definitio n?	Were the cases consecut ive?	Were the subjects compara ble?	Was the intervent ion clearly describe d?	Were the outcome measure s clearly defined, valid, reliable, and impleme nted consisten tly across all study	Was the length of follow-up adequate ?	Were the statistical methods well-describe d?	Were the results well-describe d?	

						participants?				
Patone 2021 [31]	YES	YES	YES	NO	YES	YES	YES	YES	YES	9 (Good)
Rahman 2022 [32]	YES	YES	YES	NO	YES	YES	YES	YES	YES	10 (Good)
Walker 2022 [33]	YES	YES	YES	NO	YES	YES	YES	YES	YES	11 (Good)
Wan1 2022 [34]	YES	YES	YES	NO	YES	YES	YES	YES	YES	12 (Good)
Wan2 2022 [35]	YES	YES	YES	NO	YES	YES	YES	YES	YES	13 (Good)

Table S4: shows quality Assessment of included Cohort studies using (NOS) Quality Assessment Tool.

Study ID	NOS (Cohort)(Non-coded)									
	Selection				comparability	outcome			Outcome score	Overall Appraisal
	Representativeness of the exposed cohort	Selection of the non-exposed cohort	Ascertainment of Exposure	Demonstration that that outcome of interest was not present at the start of the study	comparability Of cohorts on the basis of design or analysis controlled for confounders	Assessment of outcome	was follow up long enough for outcomes to occur	Adequacy of follow up of cohorts		
Bardenheier 1 2021 [36]	*	*	*	*	*	*	*	0	7/9	Good
Bardenheier 2 2021 [37]	*	0	-	*	*	*	*	0	6/9	Good

Clemens 2022 [38]	*	*	*	*	*	*	0	0	6/9	Fair
Frontera 2021 [39]	*	*	*	0	*	*	0	0	5/9	Poor
Huh 2021 [40]	0	0	*	0	0	*	0	0	2/9	Poor
Kant 2022 [41]	*	*	0	*	0	0	*	0	4/9	Poor
Koh 2021 [42]	*	*	*	*	0	*	*	0	6/9	Poor
Lai 2022 [43]	0	*	*	*	*	*	*	0	6/9	Good
Li 2022 [44]	*	*	*	0	*	*	*	0	6/9	Good
Nasreen 2022 [45]	0	*	*	*	0	*	*	0	5/9	poor
Shibli 2021 [46]	*	0	*	*	*	*	*	0	6/9	Good
Wong 2021 [47]	0	*	*	*	*	*	*	0	6/9	Good
Xu 2022 [48]	*	*	*	0	0	*	*	0	5/9	Poor

Good quality: 3 or 4 stars in selection domain AND 1 or 2 in Comparability domain AND 2 or 3 stars in outcome domain, **Fair quality:** 2 stars in selection domain AND 1 or 2 in Comparability domain AND 2 or 3 stars in outcome domain, **Poor quality:** 0 stars in selection domain AND 0 in Comparability domain AND 0 or 1 stars in outcome domain

Table S5: shows quality Assessment of included Case-control studies using (NOS) Quality Assessment Tool.

Study ID	NOS (Case-control) Coded									
	Selection				comparability	outcome			Outcome score	Overall Appraisal
	Is the case definition adequate ?	Representativeness of cases	Selection of Controls	Definition of controls	comparability of cases and controls on the basis of design or analysis	Assessment of outcome	was follow up long enough for outcomes to occur	Adequacy of follow up of cohorts		

					controlled for confounders					
Noseda 2021 [49]	*	*	0	*	*	*	*	0	6/9	Good

Good quality: 3 or 4 stars in selection domain AND 1 or 2 in Comparability domain AND 2 or 3 stars in outcome domain, **Fair quality:** 2 stars in selection domain AND 1 or 2 in Comparability domain AND 2 or 3 stars in outcome domain, **Poor quality:** 0 stars in selection domain AND 0 in Comparability domain AND 0 or 1 stars in outcome domain

Table S6: shows quality Assessment of included cross sectional studies using (NOS) Quality Assessment Tool.

Study ID	NOS (Cross-sectional) (non-coded)				
	Selection	comparability	outcome	Outcom	Overall

	Representativeness of the sample	sample size	Non-responsents	Ascertainment of the exposure (risk factor)	The subjects in different outcome groups are comparable, based on the study design and analysis . Confounding factors are controlled	Assessment of outcome	Statistical test	e score	Appraisal
El-Shitany1 2022 [50]	*	*	0	**	*	*	*	7/8	Good
El-Shitany2 2022 [51]	*	*	0	**	*	*	*	7/8	Good
Mohammed 2021 [52]	*	*	0	**	*	*	*	7/8	Good

Good quality: 3 or 4 stars in selection domain AND 1 or 2 in Comparability domain AND 2 or 3 stars in outcome domain, **Fair quality:** 2 stars in selection domain AND 1 or 2 in Comparability domain AND 2 or 3 stars in outcome domain, **Poor quality:** 0 stars in selection domain AND 0 in Comparability domain AND 0 or 1 stars in outcome domain

References

1. Burrows, A.; Bartholomew, T.; Rudd, J.; Walker, D. Sequential contralateral facial nerve palsies following COVID-19 vaccination first and second doses. *BMJ Case Rep.* **2021**, *14*, doi:10.1136/bcr-2021-243829.
2. Cellina, M.; D'Arrigo, A.; Floridi, C.; Oliva, G.; Carrafiello, G. Left Bell's palsy following the first dose of mRNA-1273 SARS-CoV-2 vaccine: A case report. *Clin. Imaging* **2022**, *82*, 1–4, doi:10.1016/j.clinimag.2021.10.010.
3. Čenščák, D.; Ungermann, L.; Štětkářová, I.; Ehler, E. Guillan-Barré Syndrome after First Vaccination Dose against COVID-19: Case Report. *Acta Medica (Hradec Kralove)* **2021**, *64*, 183–186, doi:10.14712/18059694.2021.31.
4. Cirillo, N.; Orlandi, M.; Colella, G. Patients with post-COVID-19 vaccination facial palsy: To boost or not to boost? *Transl. Neurosci.* **2022**, *13*, 236–239, doi:10.1515/tnsci-2022-0240.
5. Colella, G.; Orlandi, M.; Cirillo, N. Bell's palsy following COVID-19 vaccination. *J. Neurol.* **2021**, *268*, 3589–3591, doi:10.1007/s00415-021-10462-4.
6. Ercoli, T.; Lutzoni, L.; Orofino, G.; Muroni, A.; Defazio, G. Functional neurological disorder after COVID-19 vaccination. *Neurol. Sci.* **2021**, *42*, 3989–3990, doi:10.1007/s10072-021-05504-8.
7. Finsterer, J. Exacerbating Guillain-Barré Syndrome Eight Days after Vector-Based COVID-19

Vaccination. *Case Rep. Infect. Dis.* **2021**, *2021*, 3619131, doi:10.1155/2021/3619131.
8. Iftikhar, H.; Noor, S.M.U.; Masood, M.; Bashir, K. Bell's Palsy After 24 Hours of mRNA-1273 SARS-CoV-2 Vaccine. *Cureus* **2021**, *13*, e15935, doi:10.7759/cureus.15935.
9. Introna, A.; Caputo, F.; Santoro, C.; Guerra, T.; Ucci, M.; Mezzapesa, D.M.; Trojano, M. Guillain-Barré syndrome after AstraZeneca COVID-19-vaccination: A causal or casual association? *Clin. Neurol. Neurosurg.* **2021**, *208*, 106887, doi:10.1016/j.clineuro.2021.106887.
10. Ish, S.; Ish, P. Facial nerve palsy after COVID-19 vaccination - A rare association or a coincidence. *Indian J. Ophthalmol.* **2021**, *69*, 2550–2552, doi:10.4103/ijo.IJO_1658_21.
11. Manea, M.M.; Dragoș, D.; Enache, I.; Sirbu, A.G.; Tuta, S. Multiple cranial nerve palsies following COVID-19 vaccination-Case report. *Acta Neurol. Scand.* **2022**, *145*, 257–259, doi:10.1111/ane.13548.
12. Martin-Villares, C.; Vazquez-Feito, A.; Gonzalez-Gimeno, M.J.; de la Nogal-Fernandez, B. Bell's palsy following a single dose of mRNA SARS-CoV-2 vaccine: a case report. *J. Neurol.* **2022**, *269*, 47–48, doi:10.1007/s00415-021-10617-3.
13. Mason, M.C.; Liaqat, A.; Morrow, J.; Basso, R.; Gujrati, Y. Bilateral Facial Nerve Palsy and COVID-19 Vaccination: Causation or Coincidence? *Cureus* **2021**, *13*, e17602, doi:10.7759/cureus.17602.
14. Mussatto, C.C.; Sokol, J.; Alapati, N. Bell's palsy following COVID-19 vaccine administration in HIV+ patient. *Am. J. Ophthalmol. Case Rep.* **2022**, *25*, 101259, doi:10.1016/j.ajoc.2022.101259.
15. Nasuelli, N.A.; De Marchi, F.; Cecchin, M.; De Paoli, I.; Onorato, S.; Pettinaroli, R.; Savoini, G.; Godi, L. A case of acute demyelinating polyradiculoneuropathy with bilateral facial palsy after ChAdOx1 nCoV-19 vaccine. *Neurol. Sci.* **2021**, *42*, 4747–4749, doi:10.1007/s10072-021-05467-w.
16. Nishizawa, Y.; Hoshina, Y.; Baker, V. Bell's palsy following the Ad26.COV2.S COVID-19 vaccination. *QJM* **2021**, *114*, 657–658, doi:10.1093/qjmed/hcab143.
17. Obermann, M.; Krasniqi, M.; Ewers, N.; Fayad, J.; Haeberle, U. Bell's palsy following COVID-19 vaccination with high CSF antibody response. *Neurol. Sci.* **2021**, *42*, 4397–4399, doi:10.1007/s10072-021-05496-5.
18. Pothiawala, S. Bell's Palsy After Second Dose of Moderna COVID-19 Vaccine: Coincidence or Causation? *Acta Med. Litu.* **2021**, *28*, 298–301, doi:10.15388/Amed.2021.28.2.7.
19. Poudel, S.; Nepali, P.; Baniya, S.; Shah, S.; Bogati, S.; Nepal, G.; Ojha, R.; Edaki, O.; Lazovic, G.; Kara, S. Bell's palsy as a possible complication of mRNA-1273 (Moderna) vaccine against COVID-19. *Ann Med Surg (Lond)* **2022**, *78*, 103897, doi:10.1016/j.amsu.2022.103897.
20. Repajic, M.; Lai, X.L.; Xu, P.; Liu, A. Bell's Palsy after second dose of Pfizer COVID-19 vaccination in a patient with history of recurrent Bell's palsy. *Brain Behav. Immun. Health* **2021**, *13*, 100217, doi:10.1016/j.bbih.2021.100217.
21. Shalabi, F.; Lossos, A.; Karussis, D. A case report of unilateral cervical lymphadenopathy and multiple cranial neuropathies following mRNA-COVID-19 vaccination. *BMC Neurol.* **2022**, *22*, 369, doi:10.1186/s12883-022-02900-1.
22. Yu, B.-Y.; Cen, L.-S.; Chen, T.; Yang, T.-H. Bell's palsy after inactivated COVID-19 vaccination in a patient with history of recurrent Bell's palsy: A case report. *World J. Clin. Cases* **2021**, *9*, 8274–8279, doi:10.12998/wjcc.v9.i27.8274.
23. Zhang, H.; Sanchez Gomez, D.; Repajic, M.; Liu, A.K. Another Case of Bell's Palsy Recurrence After Pfizer-BioNTech COVID-19 Vaccination. *Cureus* **2022**, *14*, e27422, doi:10.7759/cureus.27422.

24. Andreozzi, V.; D'arco, B.; Pagliano, P.; Toriello, A.; Barone, P. Bilateral facial palsy after COVID-19 vaccination. *Neurol. Sci.* **2022**, *43*, 4069–4079, doi:10.1007/s10072-022-05982-4.
25. Castiglione, J.I.; Crespo, J.M.; Lecchini, L.; Silveira, F.O.; Luis, M.B.; Cotti, N.; Simison, C.J.; Aguirre, F.; Piedrabuena, M.A.; Alonso, R.N.; Azcona, C.L.; Sosa, P.S.; Maldonado, E.; Barroso, F. Bilateral facial palsy with paresthesias, variant of Guillain-Barré syndrome following COVID-19 vaccine: A case series of 9 patients. *Neuromuscul. Disord.* **2022**, *32*, 572–574, doi:10.1016/j.nmd.2022.05.003.
26. Corrêa, D.G.; Cañete, L.A.Q.; Dos Santos, G.A.C.; de Oliveira, R.V.; Brandão, C.O.; da Cruz, L.C.H. Neurological symptoms and neuroimaging alterations related with COVID-19 vaccine: Cause or coincidence? *Clin. Imaging* **2021**, *80*, 348–352, doi:10.1016/j.clinimag.2021.08.021.
27. Hamid, R.; Korkmazer, B.; Kochan Kizilkilic, E.; Arman, G.M.; Ozogul, M.; Kargın, O.A.; Arslan, S.; Kizilkilic, O. Potential Association Between COVID-19 Vaccination and Facial Palsy: Three Cases With Neuroimaging Findings. *Ear Nose Throat J.* **2022**, 1455613221113818, doi:10.1177/01455613221113818.
28. James, J.; Jose, J.; Gafoor, V.A.; Smita, B.; Balaram, N. Guillain-Barré syndrome following ChAdOx1 nCoV-19 COVID-19 vaccination: A case series. *Neurol. Clin. Neurosci.* **2021**, *9*, 402–405, doi:10.1111/ncn3.12537.
29. Mirmosayyeb, O.; Barzegar, M.; Rezaei, M.; Baharlouie, N.; Shaygannejad, V. Bell's palsy after Sputnik V COVID-19 (Gam-COVID-Vac) vaccination. *Clin. Case Rep.* **2022**, *10*, e05468, doi:10.1002/ccr3.5468.
30. Shemer, A.; Pras, E.; Einan-Lifshitz, A.; Dubinsky-Pertzov, B.; Hecht, I. Association of COVID-19 Vaccination and Facial Nerve Palsy: A Case-Control Study. *JAMA Otolaryngol. Head Neck Surg.* **2021**, *147*, 739–743, doi:10.1001/jamaoto.2021.1259.
31. Patone, M.; Handunnetthi, L.; Saatci, D.; Pan, J.; Katikireddi, S.V.; Razvi, S.; Hunt, D.; Mei, X.W.; Dixon, S.; Zaccardi, F.; Khunti, K.; Watkinson, P.; Coupland, C.A.C.; Doidge, J.; Harrison, D.A.; Ravanan, R.; Sheikh, A.; Robertson, C.; Hippisley-Cox, J. Neurological complications after first dose of COVID-19 vaccines and SARS-CoV-2 infection. *Nat. Med.* **2021**, *27*, 2144–2153, doi:10.1038/s41591-021-01556-7.
32. Ab Rahman, N.; Lim, M.T.; Lee, F.Y.; Lee, S.C.; Ramli, A.; Saharudin, S.N.; King, T.L.; Anak Jam, E.B.; Ayub, N.A.; Sevalingam, R.K.; Bahari, R.; Ibrahim, N.N.; Mahmud, F.; Sivasampu, S.; Peariasamy, K.M.; SAFECOVAC study group Risk of serious adverse events after the BNT162b2, CoronaVac, and ChAdOx1 vaccines in Malaysia: A self-controlled case series study. *Vaccine* **2022**, *40*, 4394–4402, doi:10.1016/j.vaccine.2022.05.075.
33. Walker, J.L.; Schultze, A.; Tazare, J.; Tamborska, A.; Singh, B.; Donegan, K.; Stowe, J.; Morton, C.E.; Hulme, W.J.; Curtis, H.J.; Williamson, E.J.; Mehrkar, A.; Eggo, R.M.; Rentsch, C.T.; Mathur, R.; Bacon, S.; Walker, A.J.; Davy, S.; Evans, D.; Inglesby, P.; McDonald, H.I. Safety of COVID-19 vaccination and acute neurological events: A self-controlled case series in England using the OpenSAFELY platform. *Vaccine* **2022**, *40*, 4479–4487, doi:10.1016/j.vaccine.2022.06.010.
34. Wan, E.Y.F.; Chui, C.S.L.; Lai, F.T.T.; Chan, E.W.Y.; Li, X.; Yan, V.K.C.; Gao, L.; Yu, Q.; Lam, I.C.H.; Chun, R.K.C.; Cowling, B.J.; Fong, W.C.; Lau, A.Y.L.; Mok, V.C.T.; Chan, F.L.F.; Lee, C.K.; Chan, L.S.T.; Lo, D.; Lau, K.K.; Hung, I.F.N.; Wong, I.C.K. Bell's palsy following vaccination with mRNA (BNT162b2) and inactivated (CoronaVac) SARS-CoV-2 vaccines: a case series and nested case-

control study. *Lancet Infect. Dis.* **2022**, *22*, 64–72, doi:10.1016/S1473-3099(21)00451-5.
35. Wan, E.Y.F.; Chui, C.S.L.; Ng, V.W.S.; Wang, Y.; Yan, V.K.C.; Lam, I.C.H.; Fan, M.; Lai, F.T.T.; Chan, E.W.Y.; Li, X.; Wong, C.K.H.; Chung, R.K.C.; Cowling, B.J.; Fong, W.C.; Lau, A.Y.L.; Mok, V.C.T.; Chan, F.L.F.; Lee, C.K.; Chan, L.S.T.; Lo, D.; Wong, I.C.K. mRNA (BNT162b2) COVID-19 vaccination increased risk of Bell's palsy: a nested case control and self-controlled case series study. *Clin. Infect. Dis.* **2022**, doi:10.1093/cid/ciac460.
36. Bardenheier, B.H.; Gravenstein, S.; Blackman, C.; Gutman, R.; Sarkar, I.N.; Feifer, R.A.; White, E.M.; McConeghy, K.; Nanda, A.; Bosco, E.; Mor, V. Adverse Events Following One Dose of mRNA COVID-19 Vaccination Among US Nursing Home Residents With and Without a Previous SARS-CoV-2 Infection. *J. Am. Med. Dir. Assoc.* **2021**, *22*, 2228–2232, doi:10.1016/j.jamda.2021.08.024.
37. Bardenheier, B.H.; Gravenstein, S.; Blackman, C.; Gutman, R.; Sarkar, I.N.; Feifer, R.A.; White, E.M.; McConeghy, K.; Nanda, A.; Mor, V. Adverse events following mRNA SARS-CoV-2 vaccination among U.S. nursing home residents. *Vaccine* **2021**, *39*, 3844–3851, doi:10.1016/j.vaccine.2021.05.088.
38. Clemens, S.A.C.; Fortaleza, C.M.C.B.; Crowe, M.; Pollard, A.; Tasca, K.I.; Grotto, R.M.T.; Martins, M.R.; Spadaro, A.G.; Barretti, P.; Verstraeten, T.; Clemens, R. Safety of the Fiocruz ChAdOx COVID-19 vaccine used in a mass vaccination campaign in Botucatu, Brazil. *Vaccine* **2022**, *40*, 6722–6729, doi:10.1016/j.vaccine.2022.08.026.
39. Frontera, J.A.; Tamborska, A.A.; Doheim, M.F.; Garcia-Azorin, D.; Gezegen, H.; Guekht, A.; Yusof Khan, A.H.K.; Santacatterina, M.; Sejvar, J.; Thakur, K.T.; Westenberg, E.; Winkler, A.S.; Beghi, E.; contributors from the Global COVID-19 Neuro Research Coalition Neurological Events Reported after COVID-19 Vaccines: An Analysis of VAERS. *Ann. Neurol.* **2022**, *91*, 756–771, doi:10.1002/ana.26339.
40. Huh, K.; Kim, Y.E.; Radnaabaatar, M.; Lee, D.H.; Kim, D.W.; Shin, S.A.; Jung, J. Estimating Baseline Incidence of Conditions Potentially Associated with Vaccine Adverse Events: a Call for Surveillance System Using the Korean National Health Insurance Claims Data. *J. Korean Med. Sci.* **2021**, *36*, e67, doi:10.3346/jkms.2021.36.e67.
41. Kant, A.; Jansen, J.; van Balveren, L.; van Hunsel, F. Description of Frequencies of Reported Adverse Events Following Immunization Among Four Different COVID-19 Vaccine Brands. *Drug Saf.* **2022**, *45*, 319–331, doi:10.1007/s40264-022-01151-w.
42. Koh, J.S.; Hoe, R.H.M.; Yong, M.H.; Chiew, H.J.; Goh, Y.; Yong, K.P.; Tu, T.M.; Chan, D.W.S.; Tan, B.Y.-Q.; Yeo, L.L.L.; Quek, A.M.L.; Siow, I.; Saini, M.; Angon, J.; Rathakrishnan, S.; Chen, G.J.; Hui, A.C.-F.; Ahmad, A.; Shwe, M.P.; Tan, K.; Umapathi, T. Hospital-based observational study of neurological disorders in patients recently vaccinated with COVID-19 mRNA vaccines. *J. Neurol. Sci.* **2021**, *430*, 120030, doi:10.1016/j.jns.2021.120030.
43. Lai, F.T.T.; Chua, G.T.; Chan, E.W.W.; Huang, L.; Kwan, M.Y.W.; Ma, T.; Qin, X.; Chui, C.S.L.; Li, X.; Wan, E.Y.F.; Wong, C.K.H.; Chan, E.W.Y.; Wong, I.C.K.; Ip, P. Adverse events of special interest following the use of BNT162b2 in adolescents: a population-based retrospective cohort study. *Emerg. Microbes Infect.* **2022**, *11*, 885–893, doi:10.1080/22221751.2022.2050952.
44. Li, X.; Raventós, B.; Roel, E.; Pistillo, A.; Martinez-Hernandez, E.; Delmestri, A.; Reyes, C.; Strauss, V.; Prieto-Alhambra, D.; Burn, E.; Duarte-Salles, T. Association between covid-19 vaccination, SARS-CoV-2 infection, and risk of immune mediated neurological events: population based cohort

and self-controlled case series analysis. *BMJ* **2022**, *376*, e068373, doi:10.1136/bmj-2021-068373.
45. Nasreen, S.; Calzavara, A.; Buchan, S.A.; Thampi, N.; Johnson, C.; Wilson, S.E.; Kwong, J.C.; Canadian Immunization Research Network (CIRN) Provincial Collaborative Network (PCN) Ontario investigators Background incidence rates of adverse events of special interest related to COVID-19 vaccines in Ontario, Canada, 2015 to 2020, to inform COVID-19 vaccine safety surveillance. *Vaccine* **2022**, *40*, 3305–3312, doi:10.1016/j.vaccine.2022.04.065.
46. Shibli, R.; Barnett, O.; Abu-Full, Z.; Gronich, N.; Najjar-Debbiny, R.; Doweck, I.; Rennert, G.; Saliba, W. Association between vaccination with the BNT162b2 mRNA COVID-19 vaccine and Bell's palsy: a population-based study. *Lancet Reg. Health Eur.* **2021**, *11*, 100236, doi:10.1016/j.lanepe.2021.100236.
47. Wong, C.K.H.; Lau, K.T.K.; Xiong, X.; Au, I.C.H.; Lai, F.T.T.; Wan, E.Y.F.; Chui, C.S.L.; Li, X.; Chan, E.W.Y.; Gao, L.; Cheng, F.W.T.; Tang, S.C.W.; Wong, I.C.K. Adverse events of special interest and mortality following vaccination with mRNA (BNT162b2) and inactivated (CoronaVac) SARS-CoV-2 vaccines in Hong Kong: A retrospective study. *PLoS Med.* **2022**, *19*, e1004018, doi:10.1371/journal.pmed.1004018.
48. Xu, S.; Hong, V.; Sy, L.S.; Glenn, S.C.; Ryan, D.S.; Morrissette, K.L.; Nelson, J.C.; Hambidge, S.J.; Crane, B.; Zerbo, O.; DeSilva, M.B.; Glanz, J.M.; Donahue, J.G.; Liles, E.; Duffy, J.; Qian, L. Changes in incidence rates of outcomes of interest in vaccine safety studies during the COVID-19 pandemic. *Vaccine* **2022**, *40*, 3150–3158, doi:10.1016/j.vaccine.2022.04.037.
49. Noseda, R.; Ripellino, P.; Ghidossi, S.; Bertoli, R.; Ceschi, A. Reporting of Acute Inflammatory Neuropathies with COVID-19 Vaccines: Subgroup Disproportionality Analyses in VigiBase. *Vaccines (Basel)* **2021**, *9*, doi:10.3390/vaccines9091022.
50. El-Shitany, N.A.; Bagher, A.M.; Binmahfouz, L.S.; Eid, B.G.; Almukadi, H.; Badr-Eldin, S.M.; El-Hamamsy, M.; Mohammedsaleh, Z.M.; Saleh, F.M.; Almuhayawi, M.S.; Alghamdi, S.A.; Arab, R.A.; Ali, S.S.; Harakeh, S.; Alghamdi, B.S. The Adverse Reactions of Pfizer BioNTech COVID-19 Vaccine Booster Dose are Mild and Similar to the Second Dose Responses: A Retrospective Cross-Sectional Study. *Int. J. Gen. Med.* **2022**, *15*, 6821–6836, doi:10.2147/IJGM.S376316.
51. El-Shitany, N.A.; Harakeh, S.; Badr-Eldin, S.M.; Bagher, A.M.; Eid, B.; Almukadi, H.; Alghamdi, B.S.; Alahmadi, A.A.; Hassan, N.A.; Sindi, N.; Alghamdi, S.A.; Almohaimeed, H.M.; Mohammedsaleh, Z.M.; Al-Shaikh, T.M.; Almuhayawi, M.S.; Ali, S.S.; El-Hamamsy, M. Minor to Moderate Side Effects of Pfizer-BioNTech COVID-19 Vaccine Among Saudi Residents: A Retrospective Cross-Sectional Study. *Int. J. Gen. Med.* **2021**, *14*, 1389–1401, doi:10.2147/IJGM.S310497.
52. Mohammed, R.A.; Garout, R.M.; Wahid, S.; Ayub, F.; Firas ZinAlddin, L.M.; Sultan, I. A Survey on the Side Effects of Pfizer/BioNTech COVID-19 Vaccine Among Vaccinated Adults in Saudi Arabia. *Cureus* **2021**, *13*, e19222, doi:10.7759/cureus.19222.
